# Wearable, wireless, multi-sensor device for monitoring tissue circulation after free-tissue transplantation: a multicentre clinical trial

**DOI:** 10.1038/s41598-022-21007-8

**Published:** 2022-10-03

**Authors:** Yoko Tomioka, Masaki Sekino, Jian Gu, Masakazu Kurita, Shuji Yamashita, Shimpei Miyamoto, Takuya Iida, Koji Kanayama, Kotaro Yoshimura, Masahiro Nakagawa, Satoshi Akazawa, Yu Kagaya, Kentaro Tanaka, Yuki Sunaga, Keiko Ueda, Takuya Kawahara, Yukiko Tahara, Mutsumi Okazaki

**Affiliations:** 1 Department of Plastic and Reconstructive Surgery, Graduate School of Medicine, The University of Tokyo, Tokyo, Japan; 2 Department of Electrical Engineering and Information Systems, Graduate School of Engineering, The University of Tokyo, Tokyo, Japan; 3 Department of Plastic Surgery, Jichi Medical University, Shimotsuke, Japan; 4 Division of Plastic and Reconstructive Surgery, Shizuoka Cancer Center Hospital, Shizuoka, Japan; 5 Division of Plastic and Reconstructive Surgery, National Cancer Center Hospital, Tokyo, Japan; 6 Department of Plastic and Reconstructive Surgery, Graduate School of Medical Sciences, Tokyo Medical and Dental University, Tokyo, Japan; 7 Clinical Research Promotion Center, The University of Tokyo Hospital, Tokyo, Japan

**Keywords:** Translational research, Biomedical engineering, Phase II trials

## Abstract

Wearable sensors have seen remarkable recent technological developments, and their role in healthcare is expected to expand. Specifically, monitoring tissue circulation in patients who have undergone reconstructive surgery is critical because blood flow deficiencies must be rescued within hours or the transplant will fail due to thrombosis/haematoma within the artery or vein. We design a wearable, wireless, continuous, multipoint sensor to monitor tissue circulation. The system measures pulse waves, skin colour, and tissue temperature to reproduce physician assessment. Data are analysed in real time for patient risk using an algorithm. This multicentre clinical trial involved 73 patients who underwent transplant surgery and had their tissue circulation monitored until postoperative day 7. Herein, we show that the overall agreement rate between physician and sensor findings is 99.2%. In addition, the patient questionnaire results indicate that the device is easy to wear. The sensor demonstrates non-invasive, real-time, continuous, multi-point, wireless, and reliable monitoring for postoperative care. This wearable system can improve the success rate of reconstructive surgeries.

## Introduction

Free microvascular tissue transplantation is indispensable in current medicine. The creation of anastomoses of the vessels of the transplant tissue and the vessels of the recipient site enables various tissues, such as cutaneous, subcutaneous, osseous, muscular, or even organs to be transplanted^1,2^. Since it was first reported in the early 1970s, free microvascular tissue transplantation has enabled both functional and aesthetic reconstruction for tissue deficits due to congenital anomalies, trauma, and malignant tumour resection^1–4^. Furthermore, vascularised allotransplantation, such as face transplants or limb transplants, has become a reality in recent decades^5,6^.

While the benefits of microvascular tissue transplantation are substantial, blood-flow deficiencies occur with an incidence rate of 3–5%^7–9^. This is due to thrombosis or other trouble of the artery and veins which the vascularisation of the graft relies on. This deficiency should be detected as soon as possible and corrected promptly by emergency surgery; otherwise, tissue injury will progress, and the graft will be lost^7–10^. Thus, evaluation of real-time tissue circulation is particularly critical in postoperative care following tissue grafting^11^. The greatest risk of blood flow deficiency is during the first week after surgery; therefore, a blood flow monitoring device must provide continuous postoperative monitoring^9,12,13^. In addition, multi-point measurements are essential because flow deficiencies do not always occur as a complete black-and-white or all-or-none event. Generally, deficiency occurs gradually, and in those cases, the sign partially appears in the early stages. Moreover, in cases when flow deficiency occurs in a branch of the vessels, the sign appears only in the following area. As such, multi-point measurements are needed for early and adequate detection. A device that is portable and comfortable to wear such that patients can ambulate freely is also important in continuous monitoring. There have been reports on various devices for tissue monitoring, but no single device reported to date can satisfy all these requirements^14–26^. Frequent medical examination is an alternative approach; however, the time and human resources required for this strategy are substantial, and assessments may vary among staff^14^. Furthermore, even with frequent medical examinations, a delay can occur between the onset of abnormal findings and the next assessment.

New techniques in fabrication and implementation enabled producing thin and wearable sensors that accurately monitor certain parameters for accurate clinical feedback^27–46^. Most studies have measured a certain single observation variable in laboratories, and even in clinical trials which have started to be reported also measure a certain parameter^47,48^. Instead of a single parameter, we applied multi-point, multi-modal measurement to meet the aforementioned demand. We decided to follow this style referring to physician’s overall judgments based on multiple observation items. We utilised our experience of ultra-flexible, thin electronic sensor arrays to design a wearable sensor durable enough for clinical application^49–52^. We have previously reported the utility and accuracy of the sensor in animal models and a healthy subject model^53,54^. By applying algorithmic analysis to assess the multi-modal data, we were able to reproduce the decisions of clinical surgeons using the sensor-based assessment^55^. Details of sensor and algorithm are described in material and methods.

We used our wearable sensor to monitor 73 patients following tissue grafting surgery in two clinical trials. The device was worn for 24 h for seven days. The high rate of agreement of the sensor system with physician assessment and the results of the questionnaire support the reliability of a wearable monitoring system after free-tissue transplantation.

This study investigates the validity of wearable devices after microvascular tissue transplantation, where accurate monitoring of tissue circulation is required. We believe this continuous, multi-point, wireless, and wearable monitoring system has a large potential to improve the success rate of autograft and allograft transplantation, improve the quality of life of patients, and improve the quality of life of medical staff.

## Results

**Figure 1:**
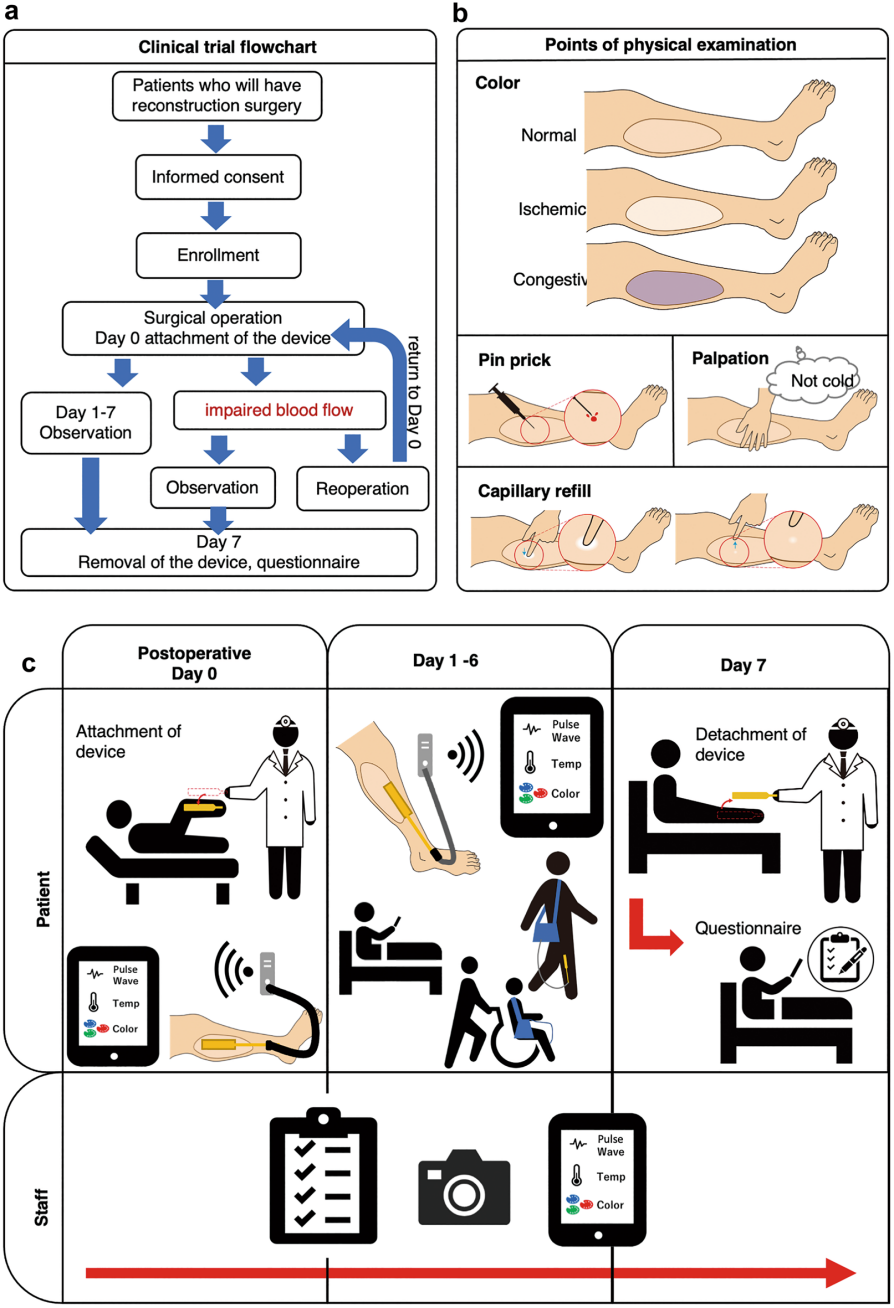
Overview of study workflow. (**a**) Flowchart of the clinical trial. Participants undergoing tissue transplant surgery provided informed consent and were fitted with the blood flow monitoring device at the graft site until postoperative day 7. (**b**) Concurrently, typical physical examinations of the graft site were performed twice daily by surgeons, as per the usual routine. Those assessments were observation of the tissue colour to determine whether it was ischemic or congestive, pin prick findings, where the tissue is pricked using a needle and the colour and speed of bleeding are observed, palpation to determine the temperature of the transplanted tissue, and a capillary refill test, which is a quick test to observe blood flow through peripheral tissue by compressing the tissue until it turns white and observing the time taken for the colour to return. (**c**) On the day participants underwent transplant surgery, the blood flow monitoring device was attached. Over 1 week, monitoring data collected from the device were transmitted to a tablet by a Bluetooth transmitter; thus, patients were not restricted from getting out of bed. Twice a day, surgeons performed the exam, recorded their findings, and provided a diagnosis of whether the tissue had sufficient circulation. Clinical photographs were also taken for comparison with data collected from the device. The device was removed on postoperative day 7 and the device site was assessed for abnormalities and clinical outcome. Patients then completed a questionnaire about their comfort and quality of life while wearing the device.

**Figure 2:**
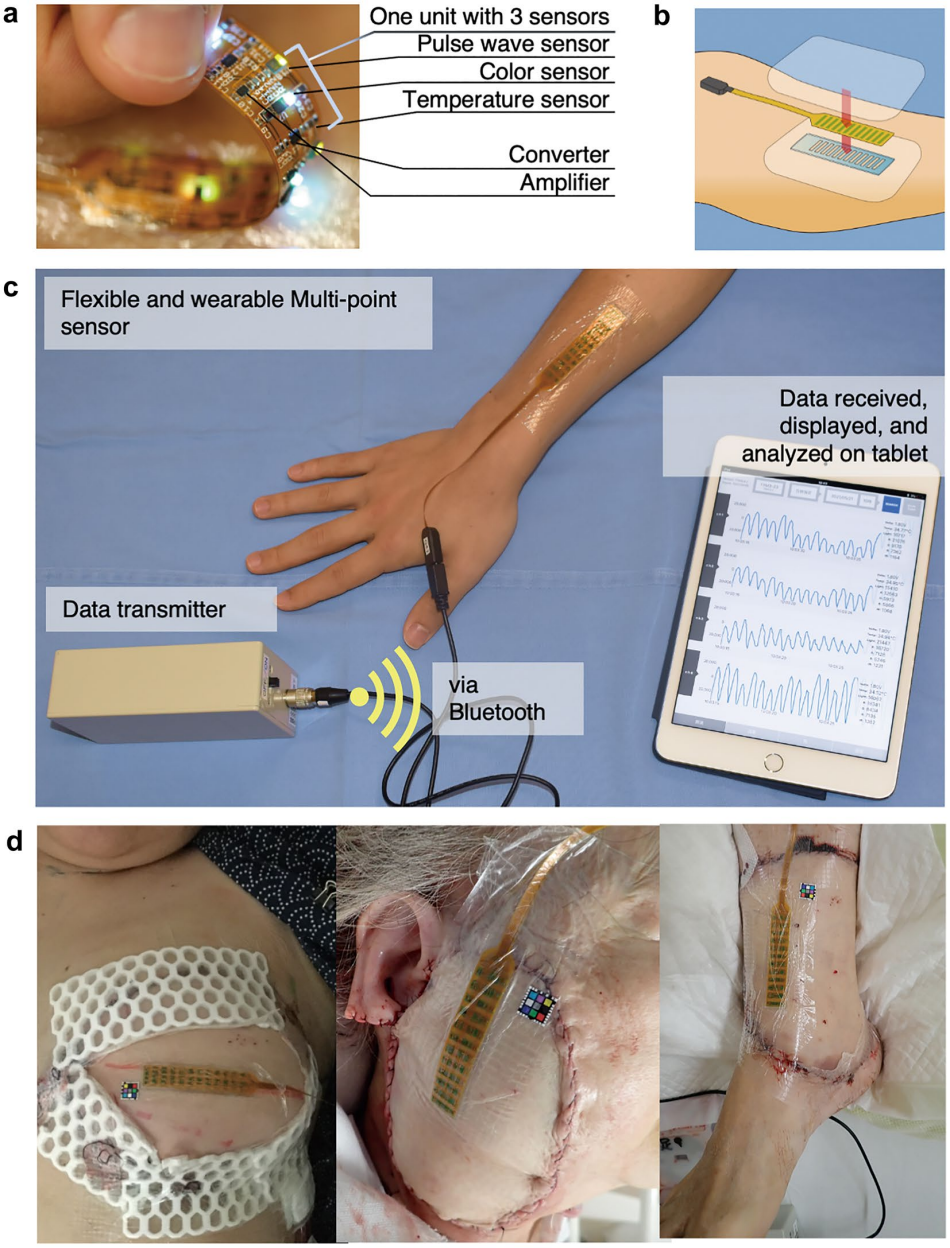
System overview. (**a**) Sensor probe details. One unit consists of three sensors which continuously monitor pulse waveforms, tissue colour, and tissue temperature. These three components were selected because the efficacy of pulse waveforms has been previously studied, and tissue colour and temperature are the key features assessed by surgeons during postoperative care in a clinical setting^14,47^. This probe is an array of units with four channels, which can be configured according to the graft size. The flexible sensor conforms to various shapes and positions on the body. (**b**) Adapting the sensor. First, a medical dressing film was placed on the skin. Then, a polydimethylsiloxane sheet was placed beneath the sensor probe to ensure the sensor remains attached despite its surface irregularities. The sensor probe was then fitted. Lastly, another medical dressing film was used to cover the sensor and keep it attached to the skin. (**c**) Overview of the system setup. Data were transmitted via Bluetooth to a tablet, where they could be displayed and analysed. We created a smartphone/tablet application to store and visualise the data collected by the sensor to enhance the device’s versatility. This would enable several physicians to receive alarms and notifications when a blood circulation issue is suspected. Moreover, they could access the data at any time by installing the application on their own device. (**d**) Flexibility supporting the wearability of the sensor. Participants included patients who underwent breast reconstruction after breast cancer treatment, head and neck reconstruction after carcinoma treatment, limb reconstruction after trauma, among others. The sensor was flexible enough to adapt to these variously curved surfaces.

**Figure 3:**
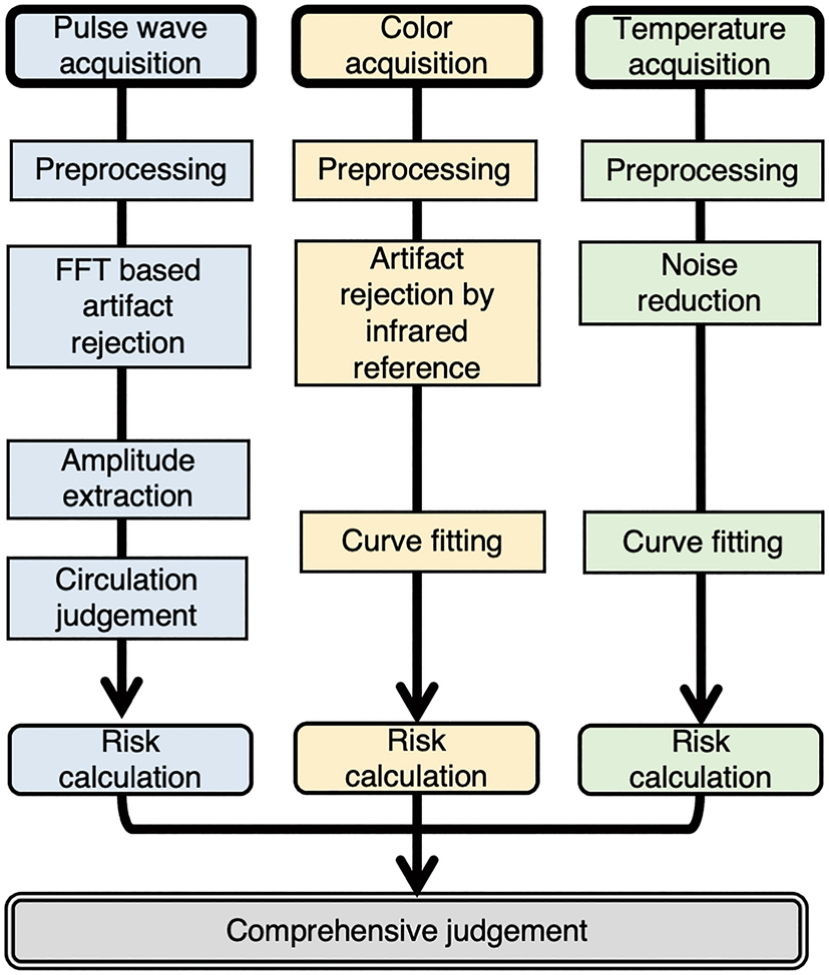
Flow chart of the algorithm for sensor-based assessment of tissue circulation. The pulse wave signal is analysed using a fast Fourier transform (FFT) to estimate fluctuations in capillary blood volume in real time. The colour and temperature signals are analysed by curve fitting to estimate the change in these parameters in the event that circulation is compromised. The risk rates are independently calculated using the algorithm described in Supplementary Fig. 2 and integrated to provide a comprehensive judgement.

The study workflow is presented in Fig. 1. Seventy-eight patients undergoing free-tissue transplantation for functional or aesthetic reconstruction after tissue loss were enrolled. We collected the physical exam findings of the physicians from patient assessments conducted during morning and evening rounds. These data were integrated with the risk rate calculated by the wearable sensor system (Fig. 2) based on algorithmic analysis of the acquired tissue temperature, pulse waveform, and colour data. The agreement between physician and wearable sensor findings was the primary outcome of this study. A flow chart of the algorithm used for risk assessment is shown in Supplementary Fig. 2. The algorithms for pulse waveform, colour, and temperature measurements were executed independently prior to calculating the overall risk score (Fig. 3).

**Figure 4:**
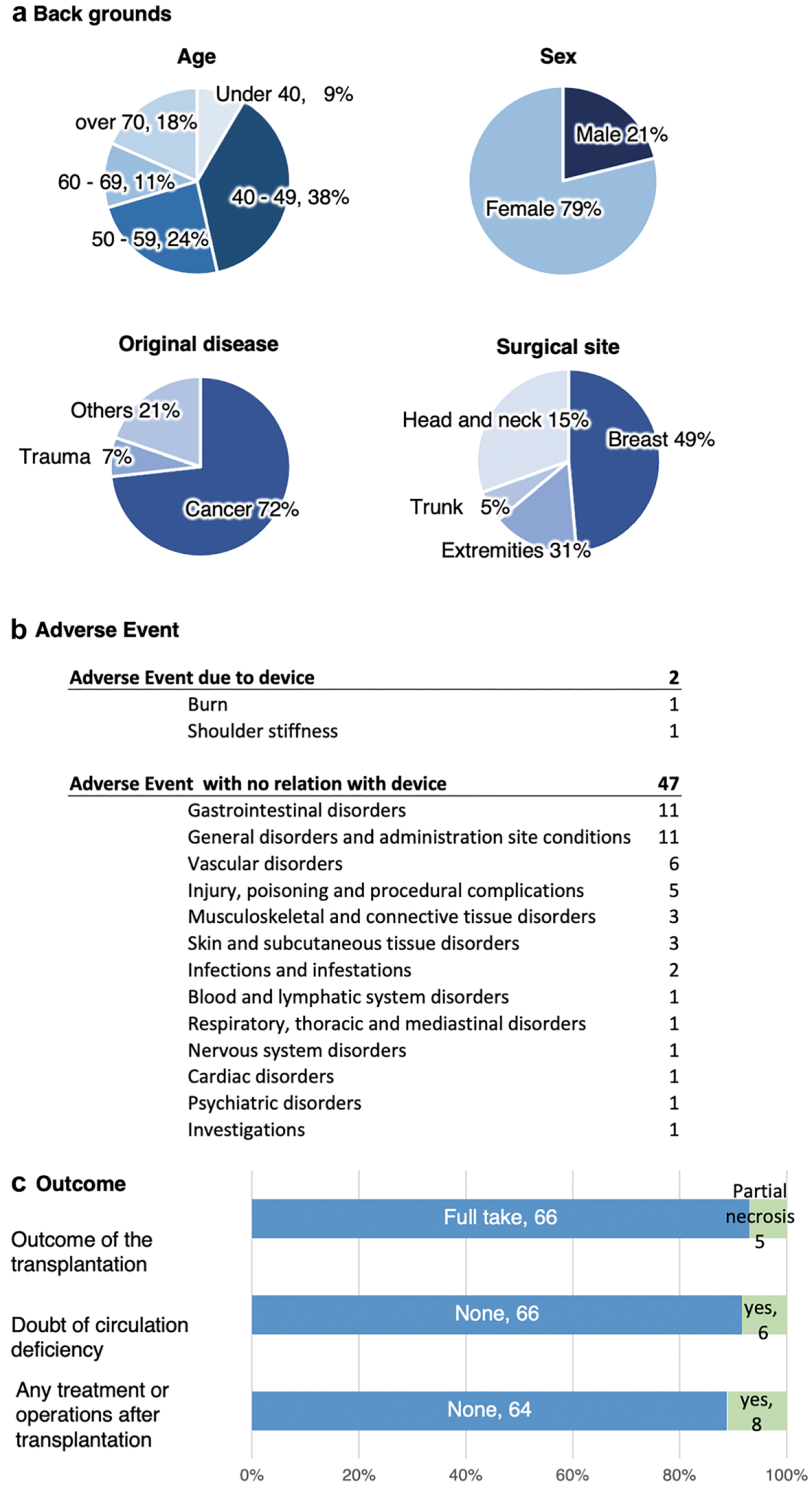
Overview of the clinical trial population—demographics and outcomes. (**a**) Breakdown of the trial population by several variables. The participants were 54.5 years of age on average, but all age categories were represented. Of these, 79% of the participants were female, reflecting the large number of breast reconstruction patients. The original disease was cancer in 73% of cases. Almost half of the surgical sites were breasts, followed by the extremities (31%), and head and neck (15%). (**b**) Summary of the adverse events experienced by patients. Unfortunately, our first case had a 1 cm × 1.5 cm burn due to insufficient waterproofing of the sensor system. After deep reflection, we improved the system by increasing the waterproofing of the sensor and implementing functions to prevent excessive current flow. One additional type of adverse event caused by the device was shoulder discomfort from prolonged wear in all cases. (**c**) Summary of tissue transplantation clinical outcomes. There was no total necrosis, partial necrosis in 7.6% of cases, and full survival of the transplant tissue in the remaining 92.4% of cases.

For the first patient registered in our clinical trial, effusion from the wounded area caused a component of the sensor to short circuit, which resulted in a 1 cm × 1.5 cm burn to the transplanted tissue. This adverse event resulted from insufficient waterproofing of the sensor system. Following this case, the clinical study was immediately cancelled, and we improved the system by increasing the waterproofing of the sensor and implementing functions to prevent excessive current flow. Once the improved safety measures were implemented (Supplementary Data “Risk management”), we sought new ethical approval for a subsequent clinical trial that enrolled 27 patients for preliminary investigations. The data from that preliminary study supported the main clinical trial described in this paper and the examination and validation of the system. We subsequently enrolled an additional 50 patients in an inspection study to evaluate the measurement performance of the sensor system after revising the trial protocol. The participants were 54.5 ± 13.0 years of age on average, and the primary cause of tissue loss was tissue resection due to cancer (73% of patients). The surgical sites were mostly breast tissue (48%), though other surgical sites including the head and neck (15%) and the trunk (6%) were evaluated (Fig. 4).

### Clinical trial 1: exploratory study

**Figure 5:**
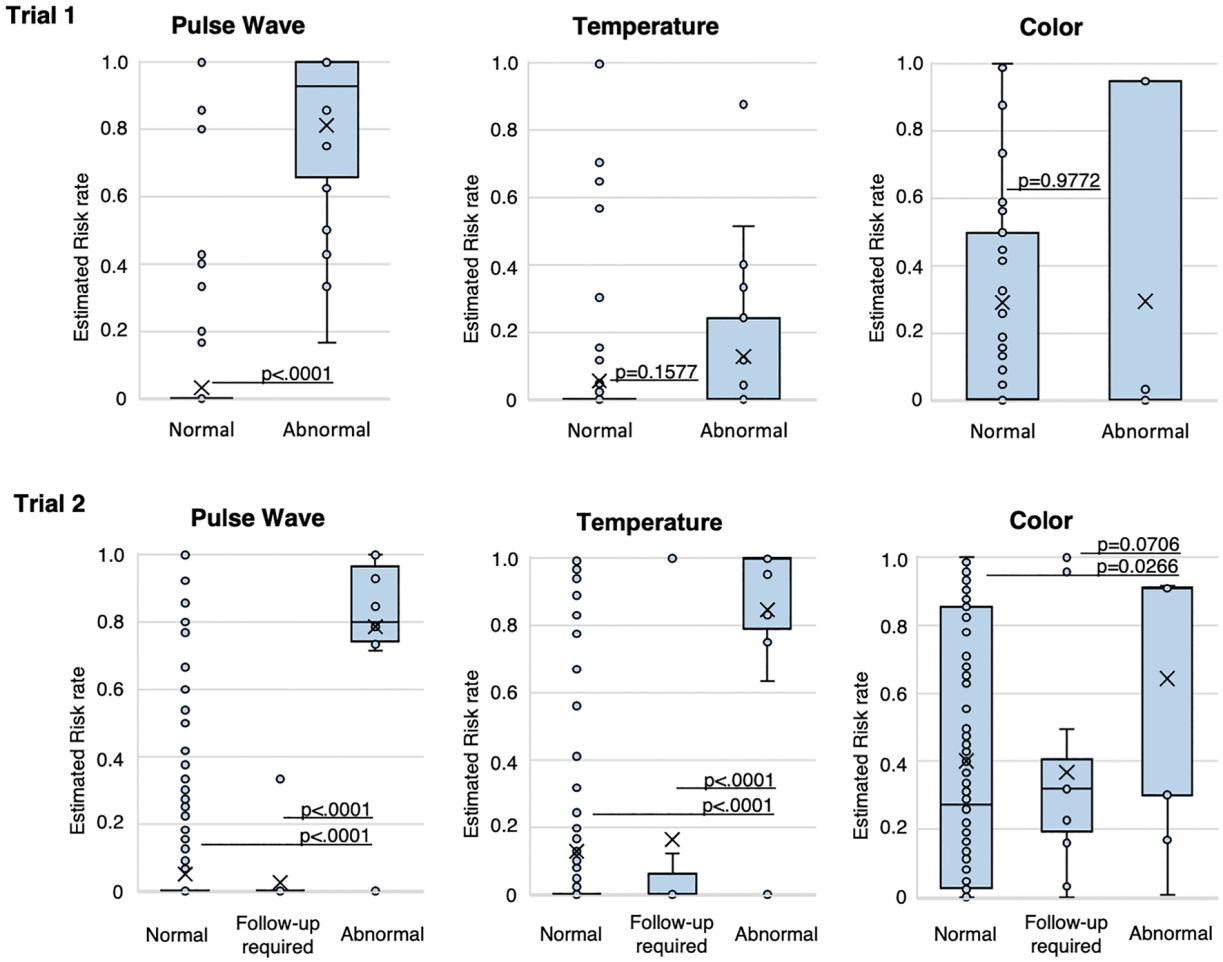
Comparison of the results from the blood flow deficiency algorithm and physician findings. The risk rate calculated for each parameter was stratified by the findings of the medical staff for each patient. A statistically significant difference was seen between the normal and abnormal cases for the pulse wave parameter in Trial 1. Because we noticed that in a clinical setting, there are some cases that are hard to determine as either normal or abnormal, in the next trial, we added follow-up required as an alternative finding for the physician. In Trial 2 (*n* = 45), a statistically significant difference was seen between the normal and abnormal cases for all three parameters (pulse wave, colour, and temperature). A significant difference was also seen between the neither normal nor abnormal (follow-up required) and the abnormal cases for the pulse wave and temperature parameters.

We enrolled 27 patients in the exploratory study conducted after improving the safety of the device. We attached our device to the graft site and recorded the data collected by the device as well as clinical findings provided by the surgeons twice a day (at each morning and evening rounds). Recording was continued until postoperative day 7, when the risk of insufficient blood flow to the graft site decreases^2,5–7^. We collected 221 total datapoints (Fig. 5) and the overall agreement between the comprehensive risk judgment by the algorithm and the judgment of the physician regarding blood flow deficiency was 96%. The rates of agreement between the physician and the algorithm for the pulse waveform, colour, and temperature features considered individually were 96.4%, 86.9%, and 69.1%, respectively.

### Clinical trial 2: device performance study

Following the initial exploratory study described above, we refined our study design and conducted Clinical Trial 2 as a device performance test. In revising our study design, we most significantly considered that the physician can have a judgment of not only ‘normal’ or ‘abnormal’ but also ‘neither normal nor abnormal’. In the ‘neither normal nor abnormal’ case, the physician’s examination reveals that the tissue does not appear definitively normal, and therefore follow-up examination is required shortly thereafter due to the risk of abnormality. Based on this, we redefined the classification of the physicians’ medical opinions to include three possible categories. A total of 56 patients were enrolled in the second trial; however, one patient withdrew consent, five did not meet the inclusion criteria, and five were unable to wear the device because of changes in the surgical technique or size of the tissue. Therefore, 45 patients were fitted with the device following surgery. We collected 526 total datapoints from these 45 patients and the rate of agreement between the comprehensive risk judgment by the algorithm and the physician judgment was 99.2% (Table 1). The rates of agreement between the physician and the algorithm when the pulse waveform, colour, and temperature were each considered individually were 93.4%, 85.5%, and 66.4%, respectively.

**Table 1:** Rate of agreement between the comprehensive risk judgment by the algorithm and the physician’s judgement.

		Staff findings			
		Normal	Follow-up required	Abnormal	Total
Comprehensive judgment as device output	Normal	497	13	1	511
	Abnormal	3	0	12	15
	Total	500	13	13	526
Rate of agreement including follow-up required				96.8% (94.9%, 98.1%)	
Rate of agreement excluding follow-up required				99.2% (98.0%, 99.8%)	

As an overall evaluation of the blood flow to the grafted tissue, the device outputs a comprehensive risk judgement calculated from the assessment of each of the three parameters as normal or abnormal. The rate of agreement between the algorithm’s judgement and the physician’s judgement was 96.8% overall when the follow-up required category was considered as abnormal. The rate of agreement was 99.2% when cases judged as neither normal nor abnormal (follow-up required) were excluded.

### Case presentation with no circulation deficiency

**Figure 6:**
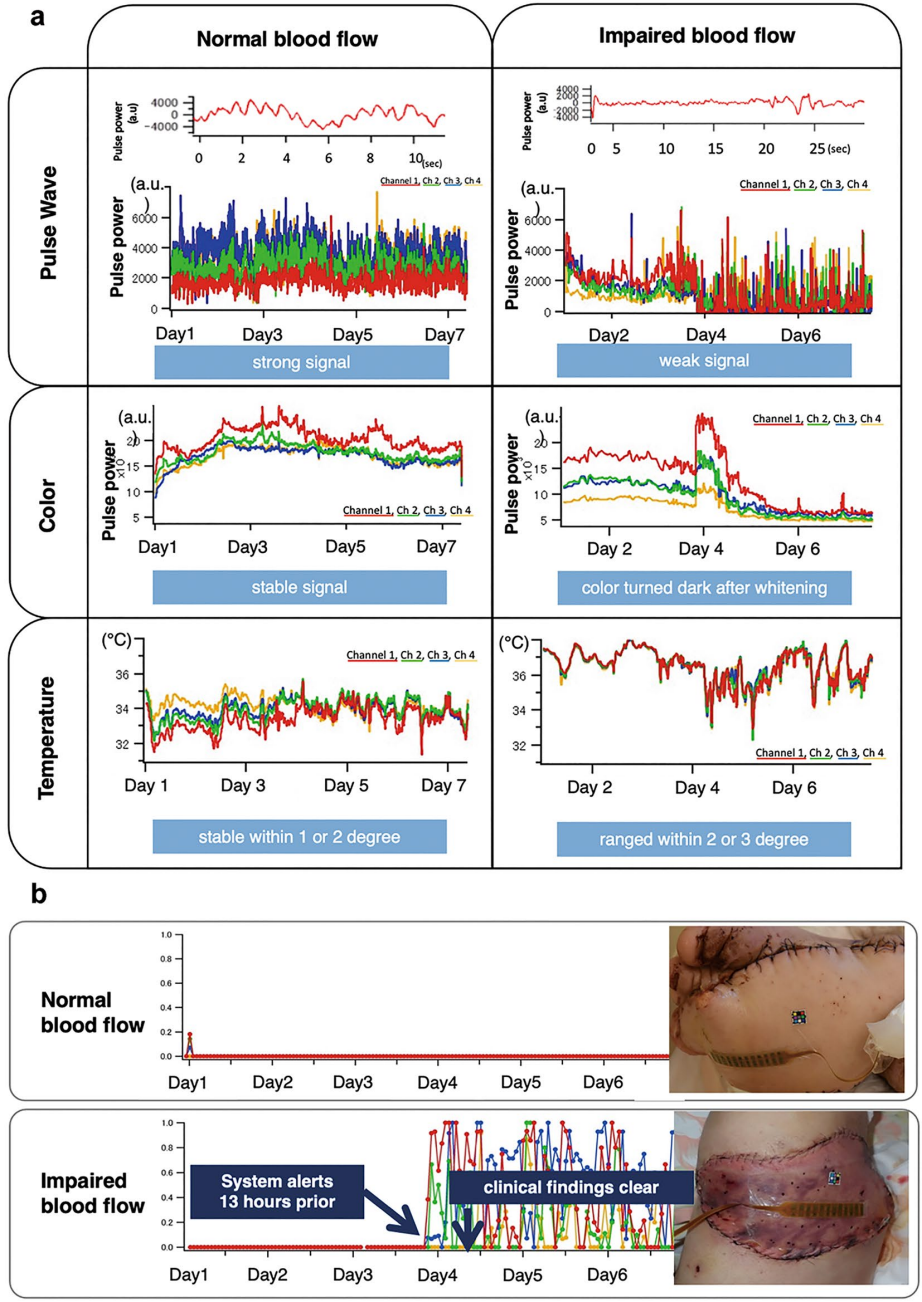
Representative case presentation of a patient with and without impaired blood flow. (**a**) Raw signal data for each measured parameter. In the case where the tissue had normal blood flow (left), the pulse wave signal was strong, the colour signal was stable, and the temperature was stable within 1° or 2°. In the case of impaired blood flow (right), the pulse wave signal was weak, the colour signal varied substantially, and the temperature varied by 2°–3°. (**b**) Comprehensive risk rate calculated from the raw data. In the normal case (top), the risk rate was close to zero throughout the postoperative week. However, in the case which tissue circulation was compromised (bottom), the calculated risk rate increased in all four sensor channels. The system identified the risk of circulation deficiency 13 h prior to medical staff reporting the change.

In Fig. 6a (left), the raw signal data for each measured parameter are shown. The pulse wave signal was strong, the colour signal was stable, and the temperature was stable. In Fig. 6b (top), we show the time course of the comprehensive risk rate for a patient without circulation deficiencies in the grafted tissue. The risk rate was calculated from the measurement of the three parameters by each of the four sensor channels and subsequent algorithmic analysis. The risk rate was consistently low throughout the week of monitoring, which was in agreement with the physician assessment as normal findings.

### Case presentation with blood circulation deficiency

In Fig. 6a, we show the raw data of each parameter. Figure 6b shows the time course of the comprehensive risk rate for a patient who experienced circulation deficiencies in the grafted tissue. In this case, the risk rate was elevated in each of the four sensor channels after approximately 7:00 p.m. on postoperative day 3. Specifically, the pulse wave output was attenuated, and the colour data showed that the graft colour became much lighter and then steadily darkened. There were no notable findings in the temperature measurements. The graft circulation was diagnosed as abnormal based on the clinician findings after 8:00 a.m. on postoperative day 4. Thus, the device detected the tissue circulation deficiency 13 h earlier than the medical staff.

### Patient questionnaire

**Figure 7:**
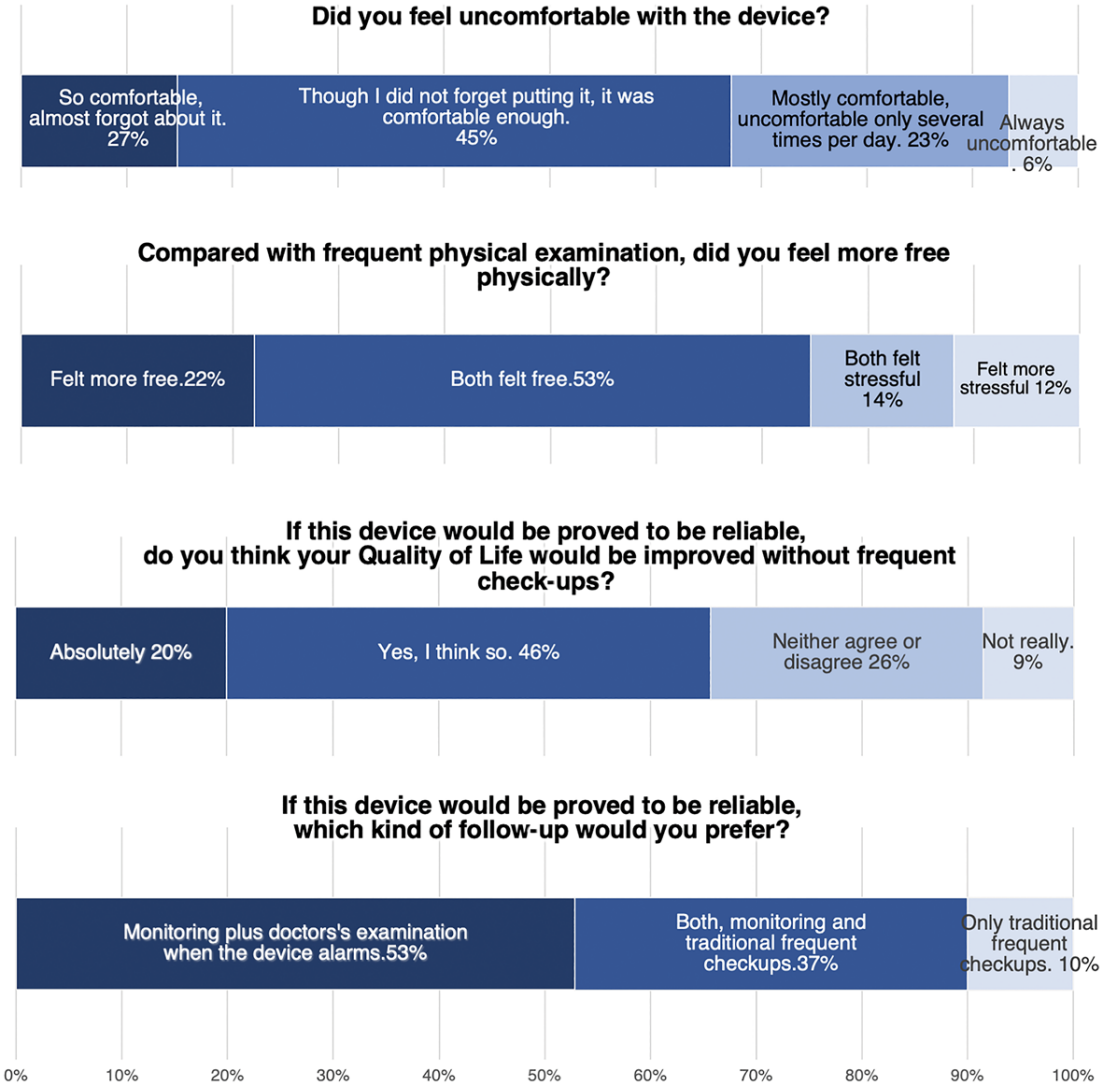
Results of patient questionnaire. On the last day of the trial, participants were asked to respond to a questionnaire. In the questionnaire, 94% of the patients responded that the wearable monitoring sensor was at least mostly comfortable, and 90% reported that they would choose to be monitored with this device in the future if needed.

At the end of the final day of the study, patients received a questionnaire to comment on their experiences after wearing the device for one week postoperatively. Patients noted that the discomfort of wearing the device was tolerable, and 90% of patients indicated that they would use this device in the future if needed (Fig. 7).

## Discussion

In our clinical trials, we showed that our wearable sensor could be used safely and effectively in clinical settings for evaluating tissue circulation following free-tissue transplantation. Importantly, the system was shown to be accurate, as the rate of agreement between the physician examination findings and the risk rate calculated by our wearable system was 99.2%. This high agreement supports the efficacy of the device for this application and suggests that other applications of wearable sensors in clinical settings should be investigated in subsequent work. The results from the questionnaire also revealed that this device was truly ‘wearable’, which is another important factor in continuous monitoring systems.

The technology for manufacturing flexible electronic devices or miniature sensors has progressed significantly in recent years^27–46^. Thus far, their efficacy has mainly been demonstrated in high-performance laboratories, since increasing the fabrication yield and durability of these devices is essential in their development toward future use in clinical settings. Recently, clinical trials have also been reported on devices that monitor activities or measure ECG signals and other vital signs^47,48,56–58^. Our device was fabricated with the sensors and circuit elements assembled on a thin, flexible substrate based on our previous studies^49–54^. In addition to its flexibility, its durability and biological safety were designed and verified for one-week use^54^. Thus, the device was easily fixed on various locations of the body for one week without discomfort, allowing flexibility throughout the clinical trial. Furthermore, we designed the device to assess tissue circulation and output results that meet the performance of a surgeon’s diagnosis. We have used the latest technology in wearable sensors to meet the clinical needs in postoperative care.

To further investigate the wearable monitoring system and definitively determine whether it facilitates early diagnosis of blood-flow abnormalities and/or improved success rates of graft repairs and blood flow restoration, a much larger trial would be crucial because of the low incidence rate of blood flow abnormalities in this context. However, because the early detection of blood flow deficiencies is known to improve success rates^9,12,25,26^, the high agreement rate of the device’s judgement and the diagnosis of the surgeons indicate that our device will contribute to the early detection of blood flow deficiencies and consequently lead to the success of transplant surgeries. In fact, in one model case, the device was able to detect blood flow deficiency 13 h prior to detection by medical staff. In this case, the blood flow deficiency of the transplanted tissue was discovered during the morning round, but the device had detected the abnormality the night before. This case highlights the value of 24-h continuous monitoring, even when patients are resting, even when there are few staff, and even when the lights are low.

Nevertheless, there are certain limitations of this study that should be acknowledged. First, the device judgement was compared with the diagnosis of the surgeons at each observation, not with whether there was an actual blood flow deficiency. This is because blood flow deficiency is obvious when the flap necrotises; however, there is no way to determine when exactly the deficiency started. Hence, during the early stage of the moderate case mentioned above, the judgement of the device and the surgeons’ diagnoses do not match. Another limitation is that because this study included 72 participants, as the size of the data set increases, the threshold that maximises sensitivity and specificity could change. We consider that by establishing a deep learning system on the cloud in the future, real data obtained by the real clinical world can be utilised to optimise the threshold value. This approach could realise a medical device that continues to innovate automatically even after it has been placed on the market.

In this study, we presented an important and feasible clinical application of flexible devices and demonstrated their safety, durability, and effectiveness. Questionnaire indicated that the wearability of the device was acceptable. Continuous, multi-point monitoring and analysis reproduced a surgeon’s assessment with a high rate of agreement.

Clinical surgeons have a clear idea of what could meet medical needs, whereas engineers have a clear idea of what is possible. Their collaboration could help them share their expertise, which could be a challenging process, but can enable us to develop something that is truly useful. Future technical developments in the manufacturing process and wireless power transfer capabilities will enable us to further enhance the usability of the device and potentially expand the applications of these devices in other clinical settings.

## Material and methods

### Ethics approval

The clinical trial protocol was approved by the Ethical Review Board of The University of Tokyo for Trial 1 (registered as UMIN000028399, 27/7/2017) and the Clinical Research Review Board of The University of Tokyo for Trial 2 (registered as jRCTs032190011, 16/4/2019). Informed written consent was obtained from all participants, and the tenets of the Declaration of Helsinki and Clinical Trials Act of Japan were followed throughout the study. This study was part of the trial and used only data from the trial.

### Standardised procedure for recording of medical findings

The clinical data in this study were collected using the EDC system (Viedoc™). The data were analysed after the observation period was completed in all cases and the data were locked. This study was also subjected to monitoring and inspection.

### Participant experience

The study physician provided an approved explanatory document and consent form to potential study patients and provided an oral explanation of the document and the study overall (Fig. 1). If the patient was deemed to have sufficient understanding of the process, an agreement was signed by free will to consent to participation in the trial. In addition, consent was obtained from the parents of one potential study subject younger than 20 years of age. Patients were not pressured during the explanation of the study or at any other time and were able to withdraw consent at any time without consequence. Patients underwent tissue transplant surgery and were subsequently fitted with a monitoring device at the transplant site when they returned to the wards. While the patients were lying in bed, the transmitter was on the bed. Patients were asked to carry the transmitter with them when they left the bed. The instrument was attached until postoperative day 7, and the data were sent to a tablet via a Bluetooth transmitter. Concurrently, diagnosis of tissue circulation deficiency was made based on clinical findings such as tissue colour, skin temperature, pin prick findings, and the presence or absence of putrescence. These parameters were measured by surgeons twice a day and recorded on predetermined record sheets and by clinical photographs. The device was removed at postoperative day 7 and the sensor site was examined by surgeons to confirm the diagnosis of blood flow deficiency and to determine whether there were any abnormalities at the graft site due to the device. Finally, the patients completed a questionnaire regarding their experience with the device.

### Statistics and reproducibility

Since Clinical Trial 1 was an exploratory study and was intended to accumulate the minimum number of cases needed to shift to the assessment of device performance, we did not design an explicit number of patients. In Clinical Trial 2, which was designed to evaluate device performance, the target number of patients was set at 50. We expected to obtain the measurement results from the device and physician findings at the same time at least 8 time points per case, and we designed a total of 400 time points from the 50 cases. The reason for this was that if a total of 400 time points of data could be obtained from 50 cases, the correlation between the judgment of the physician and the comprehensive risk judgment by the algorithm could be examined graphically. We expect an overall agreement rate of 80%, which means that if a total of 400 time points of data from 50 cases are obtained, the probability that the 95% confidence interval (one-sided) of the agreement rate will fall within 5% is more than 95%.

Statistical analysis was performed on patients for whom efficacy data were obtained at least once after fitting the study device. The frequency and proportion of patients' backgrounds were tabulated, as were the frequency and proportion of adverse events, clinical outcomes, and patient questionnaires, and presented as graphs or tables.

For each clinical trial, the percentage of agreement between and the judgment of the physician regarding blood flow deficiency was calculated to confirm the clinical utility of the algorithm. In addition, the correlation between the judgment of the physician and the comprehensive risk judgment by the algorithm was checked with a box-and-whisker plot. Analysis of variance was used to compare whether the means of comprehensive risk judgment by the algorithm differed between the judgment of the physician.

Missing value was not imputed, and multiplicity was not adjusted. All statistical tests were two-tailed and *p* < 0.05 was considered statistically significant. The statistical analysis for this paper was performed using SAS/STAT® and SAS/GRAPH® software, version 9.4 of the SAS System for Windows (SAS Institute Inc, Cary, NC, USA).

### Structure of the wearable sensor

The wearable device is comprised of a sensor, cable, data transmitter, and a tablet (as shown in Fig. 2). To obtain pulse waveform data, a reflective optical sensor (NJL5303R, Shinnihonmusen Corp, Japan) composed of a green light-emitting diode (LED; 570 nm) and a phototransistor was used for each channel. The intensity of backscattered light varies with changes in the blood volume of the capillaries, which is synchronised with the heartbeat. This phenomenon occurs because the incident light emitted from the green LED is partially absorbed when passing through blood and tissue. The temperature sensor (LMT70YFQ, Texas Instruments, USA) measured temperature by assessing the decrease in output voltage, which occurs as the temperature of a thermal diode rises. The colour sensor (P12347, Hamamatsu Photonics, Japan) detected tissue colour using a photodiode with three colours of LED (red, blue, and green) light, which irradiated the tissue and was reflected and scattered in the tissue.

After our first participant unfortunately encountered an adverse event, the waterproof coating of the device was also developed and tested^55^, demonstrating that a 30-μm-thick parylene C coating on the device could provide durability against bending, was biocompatible, and safely waterproofed the device. Safe limit of the leak current from the device to the patient body was smaller than 1 μA.

The measured values were converted to digital data on the sensor probe; then, the data were sent to the tablet via Bluetooth transmitter to be displayed and recorded. A diagnosis algorithm implemented using a monitor application was then applied to determine the state of the blood flow in the tissue by integrating the pulse wave, temperature, and colour signals. The sensor was attached to the subject with a piece of medical transparent film (Tegaderm® 3 M, USA). The outward-facing surface of the sensor was covered with an adhesive silicone sheet to flatten the sensor surface irregularities and to ensure adhesion. Finally, another medical transparent film (Tegaderm® 3 M, USA) was applied to securely fix the sensor to the transplanted tissue. This method was used to avoid having the sensor touch the skin directly, in order to reduce the risk of a potential allergic reaction.

### Information processing

#### Algorithm for pulse wave measurement

The system LEDs were turned on for 1 min and switched off for 3 min in each cycle. This conserves battery power while providing data in a timely manner, as a 3 min delay is clinically acceptable. In each cycle, data were acquired for 1 min and measured with a sampling rate of 16 Hz. During preprocessing, the algorithm segmented the 1-min data intervals into nine sampling windows. Each window in a given cycle was 16 s, with a 5 s interval between the windows. A fast Fourier transformation was applied to each sampling window and the frequency spectrum was calculated. The global peak of the spectrum from 0.5 to 2 Hz was extracted, as were local peaks with an intensity exceeding 1/3 of the global peak intensity. The frequencies of the global and local peaks in all nine windows were counted. Since the artefact measurements showed separated peak distributions and the pulse wave signals showed intensive peak distributions, the frequency at which most peaks occurred was extracted as the frequency of the pulse wave signal. Other signal artefacts were rejected. The amplitude of the pulse wave was defined as the average intensity of the corresponding extracted frequency. During circulation assessment, the amplitude of the pulse wave was compared with the threshold value. If the amplitude exceeded the threshold, the signal was judged as normal circulation in this cycle; otherwise, it was judged as compromised circulation. Finally, the pulse wave measurement was used to calculate the associated risk value. The risk value was designed to use data from the last 30 min of sampling, rather than from one data point to allow more reliable, real-time circulation monitoring^55^.

The algorithm output one of the following results every 4 min: normal circulation, compromised circulation, and motion artefact. The risk value was the ratio of the compromised circulation assessment outputs to the number of usable datapoints within the analysis period. This ratio can be calculated using the following equation:

$$ Risk_{30} = \frac{{Number_{c} }}{{Number_{c} + Number_{n} }} \times 100{\text{\% }}, $$

where $Risk_{30}$ indicates the risk value analysis over the last 30 min (approximately eight datapoints), $Number_{c}$ is the number of compromised circulation judgments, and $Number_{n}$ is the number of normal circulation judgments. $Risk_{30}$ was recalculated every 4 min. The system generated a high risk output when $Risk_{30}$ exceeded the threshold and a low risk output when $Risk_{30}$ was less than the threshold. A failed to judge output occurred when no pulse wave signal was measured due to data loss, which could occasionally occur due to loss of power or Bluetooth communication failures, but was mitigated by the multiple daily assessments.

This algorithm, which is based on frequency analysis of the signals, is computationally inexpensive and suitable for implementation in wearable devices^55^. In another paper, we report a case study where the same raw data were analysed by a different algorithm based on deep learning^59^.

#### Threshold estimation

The threshold for distinguishing normal and compromised circulation was evaluated using a receiver operating characteristic (ROC) curve, which demonstrates the relationship between the sensitivity and specificity of the sensor (Supplementary Fig. 1). The sensitivity was defined as the ratio of the number of true positive judgments of compromised blood flow to the total number of samples with compromised circulation. The specificity was defined as the ratio of the number of accurate normal judgments to the total number of samples with normal circulation. In this approach, a variable between the minimum and maximum of the data was set as a virtual threshold. The total data included the pulse power calculated from both normal and compromised circulation samples. The virtual threshold was extracted using the following conditional equation:

$$ \begin{gathered} Max \; F\left( x \right)\; = \;sensitivity\; + \;specificity - 1, \hfill \\ F\; > \;0, \;minimum \;of\; total\; data\; < \;x\; < \;maximum\; of\; total\; data, \hfill \\ \end{gathered} $$

where *x* is the virtual threshold, and the constraints showed that the threshold was effective only when *F* is greater than zero. Without this condition, the sensitivity and specificity values were too low to be verified by the approach outlined here.

#### Algorithm for colour and temperature assessment

Colour and temperature data were measured once every 4 min. The time sequence of the measurement cycle was 1 s for device setup, 0.4 s for the measurement of the temperature signal, and 1.6 s total for the measurement of the red, green, blue, and infrared signals (0.4 s for each component). The output of this algorithm was used to make an overall judgment in combination with the pulse wave algorithm output.

In clinical settings, tissue colour and temperature are expected to change gradually after venous or arterial occlusion. Thus, steep changes or fluctuations of the signal were smoothed to reduce signal noise. During preprocessing, a data set of three continuously measured points was provided as $\left[ {{ }y_{1} ,y_{2} ,y_{3} } \right]$. If $y_{2}$ was the maximum or minimum point, and three points satisfied the condition that $\left| {y_{3} - y_{2} } \right| > \left| {y_{2} - y_{1} } \right| \times 1/2$, $y_{2}$ was replaced by the value of $y_{1}$. The next three points $\left[ {{ }y_{2} ,y_{3} ,y_{4} } \right]$ were then processed. The infrared signal from the colour sensor was used to estimate the distance from the sensor to the tissue. Thus, the measurement artefact was rejected when the infrared signal changed by more than 10% of the original value. Curve fitting was also a key part of the algorithm. The colour signal transition of the tissue was assumed to be an exponential model function. Data collected over the previous 30 min were analysed, and parameters $A$ and $\tau$ were calculated using the following equation:

$$ y = A_{0} + A{*}e^{{ - \frac{{t - t_{0} }}{\tau }}} { }, $$

where $A$ represents the transition of the colour signal and $\tau$ represents the time constant of the transition. Goodness of fit ($gof$) was also calculated as a parameter for assessment of the grafted tissue. A parameter Index was defined to evaluate the change in the signal over the current window and was calculated according to the following equations:

$$ Index\; = \;gof\; \times \;lognormal\left( {\left| A \right|} \right)\; \times \;lognormal\left( \tau \right){ }, $$

$$ lognormal\left( x \right)\; = \;\frac{1}{{\sqrt {2\pi } \sigma x}}exp\left( { - \frac{{\left( {\ln x - \mu } \right)^{2} }}{{2\sigma^{2} }}} \right), $$

where $gof$ and $lognormal$ functions were normalised by dividing all data by the maximum, scaling the data to a range from 0 to 1. The $lognormal$ function was used to transform $A$ and $\tau$. Due this normalisation, the parameters should become either extremely large or small if there is no change in the signal. The $lognormal$ function yielded a greater output when the input was close to the mode value $\left( {e^{{\mu - \sigma^{2} }} } \right)$ and was approximately 0 when the input was far from the mode value. Therefore, detectable changes in the signal resulted in large function outputs, whereas a stable signal resulted in small function outputs. The $gof$ calculation used the Levenberg–Marquardt method. The parameter $\sigma$ was fixed to 0.25, and $\mu$ was set to the mode value. For analysis of brightness, the mode value was set to 1000 and for analysis of temperature, the mode value was set to 2. The risk value was provided by $Index$. For analysis of the colour signal, if the $\left| {Index} \right|$ for the last analysis window was greater than the threshold, the risk value was calculated as

$$ risk\; = \;risk_{previous} \; + \;Index{ }, $$

where $risk_{previous}$ is the risk value for the previous analysis window. Otherwise, the risk value was calculated as

$$ max\left( {risk_{previous} ,\;\left| {Index} \right|} \right){ },{ } $$

With a greater risk value, there is a higher likelihood of tissue circulation changes. The algorithm therefore provided a high risk judgment when the $risk$ value exceeded threshold; otherwise, it returned a low risk judgment. The threshold in this study was set to 0.5.

#### Comprehensive judgment

The pulse wave, colour and temperature signals were analysed and output at the end of the analysis process as risk values for each parameter (Fig. 3). Risk values were calculated for every 4 min of data and compared with the corresponding risk thresholds. The detailed process and the method of threshold estimation are outlined in Supplementary Fig. 2. The comprehensive judgment ultimately outputs a system judgment result of risky, low risk, or failed to judge, and the result is reassessed every 4 min.

The comprehensive judgment algorithm is shown in Supplementary Fig. 2. The pulse wave judgment is most consistent with clinical examination; therefore, the algorithm will yield a failed to judge result if the risk value of the pulse wave (pr) cannot be calculated. In the following steps, if the risk values of colour (cr) and temperature (tr) are not available, pr will be compared with the threshold of the pulse wave risk (pt). A risky judgment result is generated when pr is greater than pt. If tr is available, both pr and tr must be higher than the threshold risk for pulse wave and temperature, respectively, to yield a risky judgment. If cr is also available, pr, cr, and tr must all exceed their respective risk thresholds to result in a risky judgment. If a risky judgment is not reached but a pr value is present, low risk is generated as the system judgment. The risk values range from 0 to 1, as do the thresholds of three components. The thresholds were estimated from clinical trial data. The estimation method tests all possible threshold values between 0 and 1 with a step of 0.01. Therefore, the estimated thresholds should obey the following condition: pt ∈ [0.5, 0.71] & (ct = 1.0||tt = 1.0). The estimated thresholds used here are suitable for the current clinical trial, but these values may change when the population size increases. In the future, the algorithm could be modified to optimise the thresholds online and to generate a feasible result that enables real-time tissue circulation monitoring.

## Supplementary Information

Supplementary Information 1.

Supplementary Information 2.

Supplementary Information 3.

Supplementary Information 4.

Supplementary Information 5.

## Data Availability

Source data for the main figures in the manuscript can be accessed as Supplementary Data 1, 2 and 3. Further raw datasets generated during and/or analysed during the current study are not publicly available according to Swiss human research law but are available from the corresponding author on reasonable request. Proposals with reasonable requests may be submitted to yoko1031prs@gmail.com up to 24 months following article publication.
