# Supplementary material for: Wearable, wireless, multi-sensor device for monitoring tissue circulation after free-tissue transplantation: a multicentre clinical trial
Source: Sci Rep. 2022 Oct 3;12:16532. doi: 10.1038/s41598-022-21007-8 (PMC9529918; doi:10.1038/s41598-022-21007-8)
Supplement: Supplementary file 4 — Supplementary Information 4. [file 41598_2022_21007_MOESM4_ESM.docx]

# Preclinical study

## Mechanical and electrical testing

The mechanical and electrical safety of this research equipment meets the requirements of JIS (Japanese Industrial Standards) T 0601-1:2017 "Medical electrical equipment - Part 1: General requirements for basic safety and basic performance", except for "7 Signage, labelling and documentation of ME equipment", "12.2 Usability of ME equipment", and 14 Programmable electro-medical systems" are fulfilled, with the exception of "14 Programmable electro-medical systems".

With regard to '7 Signs, labels and documentation of ME equipment' and '12.2 Usability of ME equipment', safety was ensured by explaining the usage and precautions directly to the user instead of signs, labels and documentation for this equipment. With regard to "14 Programmable electro-medical systems", no software was developed in accordance with the software life cycle process, such as JIS T 2304, but the basic purpose of this device is to monitor, and the measurement results of this device are used only for data collection and not for diagnosis. The system has a hardware current limitation mechanism to prevent overcurrents, and it has been verified that the system works as designed with the software installed, so it was concluded that the programmable electro-medical system does not pose any health hazards to the subjects and has no impact on safety.

The mechanical and electrical safety of the equipment was ensured.

## Electromagnetic compatibility testing

The electromagnetic compatibility of this equipment was tested in accordance with the requirements of "36 Electromagnetic compatibility" of JIS T 0601-1-2:2012 "Medical electrical equipment Part 1-2: General requirements for safety Electromagnetic compatibility Requirements and tests", and all items met the standard. The equipment was judged to be sufficiently electromagnetic-compatible.

## Biological safety assessment

### Approach to biological safety assessment of materials in contact and wetted

When the device is used, the sensor probe is attached on top of the coating material, so the device does not come into direct contact with the wound or other parts of the body under normal conditions of use, but as the device is continuously worn on the body surface, it is necessary to evaluate the safety of the device in case of contact with the human body. In addition, there is a possibility that body fluids such as leachate may enter through gaps in the coating material and come into contact with the silicone sheet and sensor probe. Therefore, an assessment of biological safety was carried out according to JIS-T-0993-1 'Biological evaluation of medical devices - Part 1: Evaluation and testing in the risk management process'.

The materials that may come into contact or come in contact with liquid in this equipment are Parylene C from KISCO Corporation, which is used for the surface coating of the sensor probe, and Silicone Rubber-F15 from Fuji Systems Corporation, which is used for the silicone sheet.

The items to be evaluated for these materials were determined in accordance with Annex A of JIS-T-0993-1. The category of this equipment is "surface contact equipment", the contact site is "damaged surface" and the contact time is "short to medium term (>24 hours and up to 30 days)". Under these conditions, the three items "cytotoxicity", "sensitisation" and "irritation or intradermal reaction" need to be evaluated for the material concerned. The other items "systemic toxicity", "subacute and subchronic toxicity", "genotoxicity", "implantation test", "haematocompatibility", "chronic toxicity", "carcinogenicity", "reproductive and developmental toxicity", "in vivo degradation", "toxicokinetics test" and "immunotoxicity" do not require evaluation in this equipment category, contact site and contact time. The following categories have been omitted because they are not required to be evaluated for this equipment category, contact site and contact time.

For parylene C, the material manufacturer KISCO Corporation has carried out an evaluation of three items: cytotoxicity, sensitisation, irritation or intradermal reaction, and issued a report stating that there were no problems in any of them (Appendix 1).

For silicone rubber-F15, the material manufacturer, Fuji Systems Ltd, has carried out an evaluation of three items: cytotoxicity, sensitisation, irritation or intradermal reaction, and issued a report stating that there were no problems in any of them.

# Risk management

## Overview of risk analysis, including identification of residual risks.

Prior to the implementation of the previous clinical study 'Validation of a tissue blood flow monitoring system using flexible electronic devices', a risk analysis was carried out by the principal investigator and the person in charge, and tests such as leakage current, emission and temperature rise, and evaluation such as wearing by a healthy person were conducted.

Following the occurrence of adverse events during this clinical study, an intensive analysis of the risks derived from contact of the sensor probe with leachate from the affected area was conducted. The sensor probes were waterproofed and tests confirmed that the waterproofing had sufficient mechanical strength. As multiple safety measures, the transmitter was equipped with a mechanism to detect and stop operation if an abnormal current flowed through the sensor probe, and the control circuit was modified with a separate power source so that any abnormality in the sensor circuit would not affect the control circuit.

Furthermore, following the results of the PMDA (Pharmaceuticals and Medical Devices Agency) pre-development consultation in December 2017, a biological safety risk analysis and experimental assessment was carried out.

The residual risks of this equipment are inaccurate measurements and loss of function. The risk of patients being disadvantaged due to inaccurate measurements or loss of function is very low because in clinical studies using the device, doctors carry out conventional consultations without any reliance on the measurement results of the device. The likelihood of risk is also low due to the short duration of use of the device, which is a maximum of seven days. For these reasons, the residual risk was judged to be sufficiently small.

## Safety design considerations to prevent temperature rise in sensor probes.

From the temperature rise test results, a temperature rise of 1.6°C was observed at the wearing part due to the operation of this device.JIS T 01-1:2012 increases the maximum surface temperature of the wearing part from 41°C to 43°C through risk management, and a maximum surface temperature of 41°C or lower is considered safer.1. Considering a temperature increase of 1.6°C, it was found that the basic safety could be met if used at an ambient temperature of 39°C or lower. For use in hospitals (in an environment where the room temperature is kept constant), the risk of adverse phenomena occurring due to temperature rise is considered to be extremely low. As a further marginally safer design, a control mechanism was incorporated to stop operation when the temperature sensor in the device reaches 38.5°C.

## Considerations on multi-sensor interactions.

The sensor section contains four each of pulse wave, temperature and colour sensors. Pulse wave, temperature and colour measurements are made in a time-shared manner and not at the same time to prevent interaction between different types of sensors. The temperature is measured before pulse wave and colour measurements are taken to prevent the possibility of temperature being affected by the heat generated during operation of the pulse wave and colour sensors. As for interactions between different sensors of the same type, for the colour sensors, interactions are prevented by measuring the four colour sensors in a time-shared manner. For pulse wave sensors, data with no interaction was obtained by increasing the distance between sensors by at least 10 mm^3^ , and interaction was prevented by increasing the distance between sensors to 15 mm.

## Considerations regarding overcurrent protection mechanisms

As mentioned in the adverse event case described above, if a short circuit occurs in the sensor section, there is a risk of overcurrent flow and heat generation. The sensor section is waterproofed by a parylene coating, so short-circuits do not occur, but it is necessary to prevent heat generation due to overcurrent in case the waterproofing is damaged. The current in the sensor section is monitored during measurement, and if the specified current is exceeded, the sensor is deemed to be faulty and the power supply to the sensor section is cut off. In order for this safety mechanism to function even if the voltage of the control circuit drops due to a short circuit, the power supply of the control circuit is independent of the power supply of the sensor section, so that the safety mechanism will operate even if a short circuit occurs in the sensor section.

Furthermore, an overcurrent-prevention element similar to a breaker is incorporated in the power supply of the sensor section to provide multiple safety mechanisms.

## Biological safety considerations.

Experimental verification of the risk of wetting of electronic components and other components used in sensor probes showed that the parylene coating can function without failure within a loading range of up to a bending radius of 20 mm. Physicians and nurses participating in clinical studies should be informed in writing that the device should be used under conditions where strong bending beyond a bending radius of 20 mm is not applied. A bending radius of 20 mm is a considerably strong bending in actual use conditions, and the sensor probe cannot be used in such a way that it wraps around the finger, but it can be used without problems if it is applied to the actual site of application in the clinical studies to date.

The properties of the material may also be affected by sterilisation. The samples were EOG-sterilised, so the evaluation also takes into account the effect of sterilisation on the material.

## Safety regarding radiation.

The output of the light generated by this device is about 220 mcd (colour sensor), which is set at a level that poses no safety problems. In clinical research, the user should be informed not to bring this device into the radiotherapy room.
