# Supplementary material for: Wearable, wireless, multi-sensor device for monitoring tissue circulation after free-tissue transplantation: a multicentre clinical trial
Source: Sci Rep. 2022 Oct 3;12:16532. doi: 10.1038/s41598-022-21007-8 (PMC9529918; doi:10.1038/s41598-022-21007-8)
Supplement: Supplementary file 5 — Supplementary Information 5. [file 41598_2022_21007_MOESM5_ESM.pdf]

Supplementary information

Supplementary Figure 1. Receiver operating characteristic (ROC) curves of the pulse waveform amplitude. The red curve indicates the results calculated from the 27 cases enrolled in the first clinical trial, whereas the blue curve indicates the results calculated from the seven total cases enrolled in both clinical trials. For the analysis of data from the first trial, the F-value reached a maximum of 84.1% when the threshold was set at 846. For the analysis of data from both trials, the F-value reached a maximum of 86.4% when the threshold was set at 846.

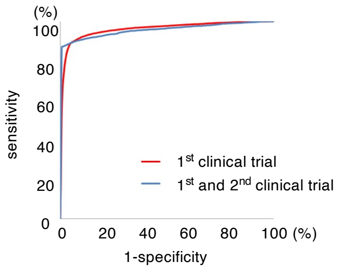

Supplementary Figure 1.

Supplementary Figure 2. Flowchart of the comprehensive judgment algorithm. Here, pr, cr, and tr represent the risk values of the pulse wave, colour, and temperature data, respectively. Further, pt, ct, and tt represent the corresponding thresholds of the pulse wave, colour, and temperature data.

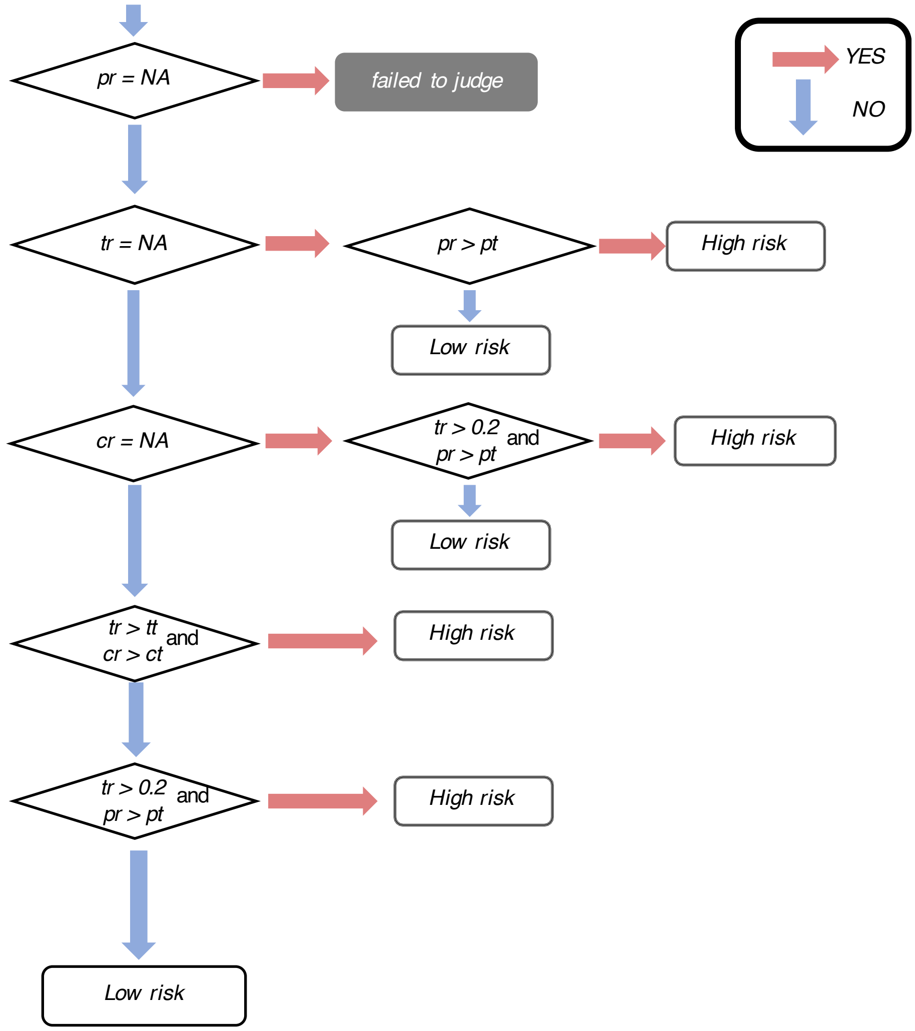

Supplementary Figure 2.
